# Light-triggered cardiac microphysiological model

**DOI:** 10.1063/5.0143409

**Published:** 2023-05-23

**Authors:** V. Vurro, K. Shani, H. A. M. Ardoña, J. F. Zimmerman, V. Sesti, K. Y. Lee, Q. Jin, C. Bertarelli, K. K. Parker, G. Lanzani

**Affiliations:** 1 Center for Nanoscience and Technology, Istituto Italiano di Teconologia, Milano, 20133 Italy; 2 Disease Biophysics Group, John A. Paulson School of Engineering and Applied Science, Harvard University, Boston, Massachusetts 02134, USA; 3 Department of Chemical and Biomolecular Engineering, Samueli School of Engineering, University of California, Irvine, California 92697, USA; 4 Department of Chemistry, Materials and Chemical Engineering “Giulio Natta” Politecnico di Milano, Milano, 20133 Italy; 5 Department of Integrative Bioscience and Biotechnology, Sejong University, Seoul, South Korea; 6 Department of Physics, Politecnico di Milano, Milano, 20133 Italy

## Abstract

Light is recognized as an accurate and noninvasive tool for stimulating excitable cells. Here, we report on a non-genetic approach based on organic molecular phototransducers that allows wiring- and electrode-free tissue modulation. As a proof of concept, we show photostimulation of an *in vitro* cardiac microphysiological model mediated by an amphiphilic azobenzene compound that preferentially dwells in the cell membrane. Exploiting this optical based stimulation technology could be a disruptive approach for highly resolved cardiac tissue stimulation.

## I. INTRODUCTION

Over the last decade, the use of optical stimulation has emerged as a powerful tool for exciting *in vitro* cardiac models and has begun to be explored as an alternative to conventional electric stimulation.^1–7^ In particular, the appeal of such an approach relies on high temporal resolution, high efficiency, and highly localized stimulation. Furthermore, the use of light as a stimulating/regulatory tool mitigates electrical wiring requirements, contact resistance, electrode placing, and material degradation that occurs in conventional systems. Due to these unique properties, photostimulation is a promising tool for research and therapeutic applications especially in the cardiovascular field and for bio-hybrid robotics.^8–17^

Because living cells and tissues are usually not sensitive to light, a suitable phototransducer has to be introduced to enable photostimulation. One of the more effective approaches has been developed by Optogenetics exploiting the expression of light sensitive channels. In particular, an exogenous DNA is carried into the cell through a viral transfection leading to the production of the light-sensitive ion channel.^18–20^ Optogenetics has been demonstrated initially in neuroscience and later on for the stimulation of cardiac^21–24^ and skeletal muscle cells.^25–32^ Despite the promising results, the required gene therapy poses doubts on its clinical applicability.^33,34^ Alternatively, one can introduce photoactive biotic–abiotic interfaces^35–48^ that transduce light into bio-electricity.^49–51^ Recently, cardiac cell photostimulation has been achieved by graphene-based interfaces,^52^ silicon nanowire,^53^ gold nanoparticles,^54^ gold nanorods,^55^ planar metasurfaces,^56^ and organic films.^57^ All these interfaces involve different triggering mechanisms that are typically capacitive, faradaic, or thermal.^58–61^ Materials and effects are characterized by advantages and drawbacks, which are discussed in a recent review.^62^

**FIG. 1.**
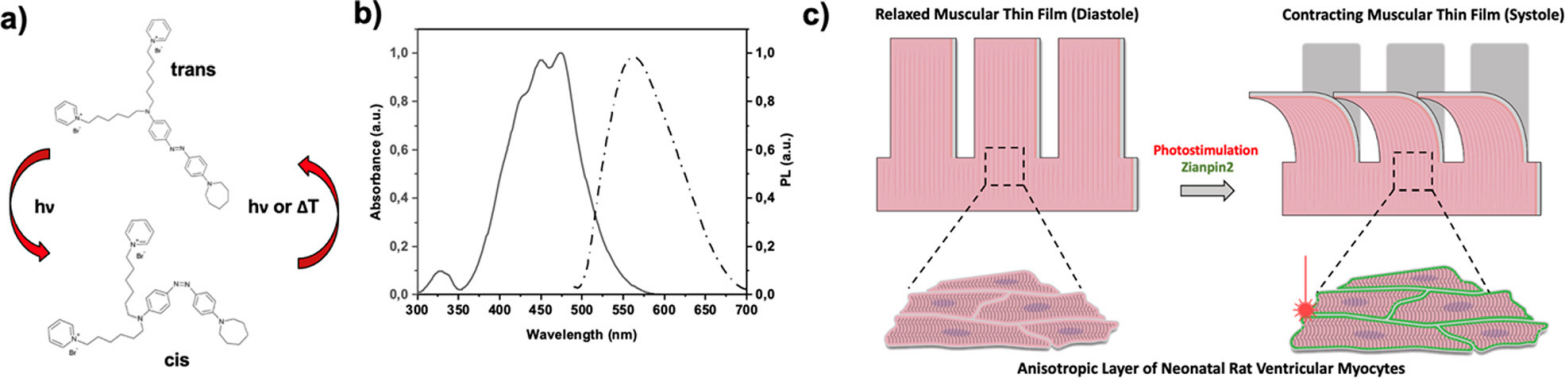
Sketch of the proposed muscular thin films (MTFs) photostimulation process. (a) Ziapin2 chemical structure and isomer spatial configuration. The image shows how trans → cis photoisomerization takes place by light excitation ( $\lambda_{ex}^{\mathrm{trans}\to \mathrm{cis}}=470\;\mathrm{nm}$) while the cist → trans process can be triggered either via optical ( $\lambda_{ex}^{\mathrm{cis}\to \mathrm{trans}}=500\;\mathrm{nm}$) or thermal processes. (b) Ziapin2 absorbance and photoluminescence spectra (Abs. is represented by a continuous line and PL by dash-dot line). (c) Sketch of an MTF treated with Ziapin2. In the inset, Ziapin2 is represented as green dots. The phototransducer is internalized by aligned neonatal rat ventricular myocytes (NRVMs) and it triggers cell contraction due to light illumination.

In this work, we present a non-genetic cardiac tissue photopacing technique based on the exploitation of an intramembrane molecular phototrigger. The proposed compound, named Ziapin2, is comprised of an aminoazobenzene core and an amphiphilic structure. Ziapin2 has been previously tested as a phototransducer initially in HEK cells in order to understand the triggering effect and to characterize the photostimulation process.^63^ Moreover, Ziapin2 has been demonstrated to be a valuable tool for neuron stimulation both *in vitro* and *in vivo*.^64^ Recently, Ziapin2 has been also used as a pacing tool with human-induced pluripotent stem cell-derived cardiomyocytes.^65^ The molecular structure, absorption, and photoluminescence spectra of Ziapin2 are reported in Fig. 1. This design enables Ziapin2 to dwell within the cell membrane and isomerize due to photoexcitation. The photoisomerization affects the cell membrane thickness and subsequently the membrane capacitance resulting into a modulation of the membrane potential leading through the usual calcium-induced calcium release process to the contraction.^63–65^ Here, in order to test Ziapin2 as a tool for photopacing cardiac tissues, we administered our phototransducers to muscular thin film (MTF) cantilevers seeded with cardiomyocytes. MTFs are microphysiological systems composed of a bio-hybrid double layer (elastic substrate and living cell layer) that can be used to quantify cellular contractile forces by measuring changes in the cantilever's radius of curvature.^66–69^ By mimicking the native properties of the heart, such as cellular organization and substrate mechanical properties, MTFs can serve as *in vitro* models for monitoring both cardiac health and disease.^70–74^

## II. RESULTS

**FIG. 2.**
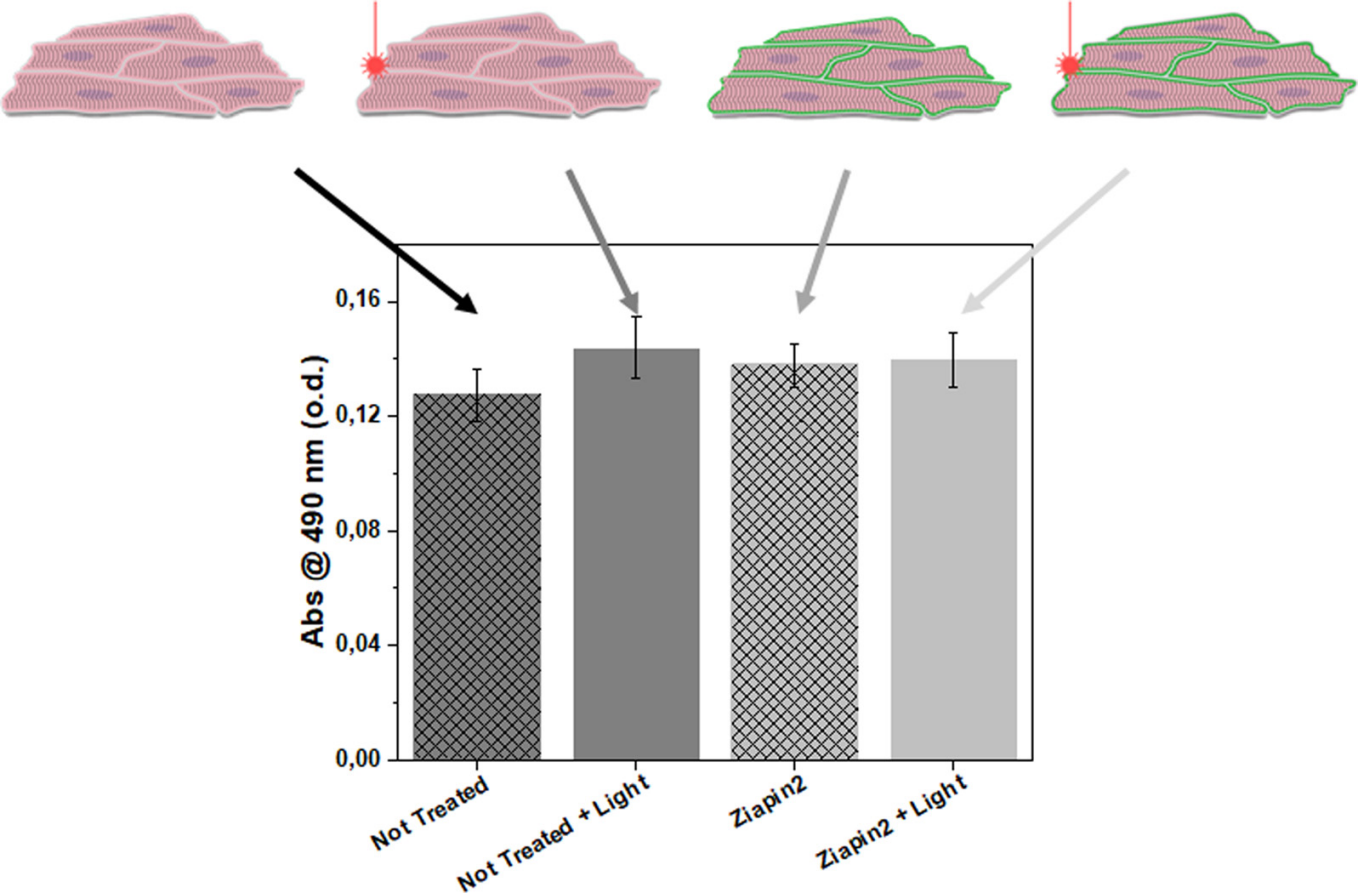
Viability assay. Effect of ZIAPIN2 internalization and light exposure on NRVMs viability measured with the MTS assay. In the histogram, the dark gray bars represent the untreated cells viability in the dark condition (patterned bar) and after the light exposition (unpatterned bar). The light gray bars represent the ZIAPIN2-treated NRVMs viability in the dark condition (patterned bar) and after the light exposition (unpatterned bar). Data are represented as mean±standard error of the mean (s.e.m.).

**FIG. 3.**
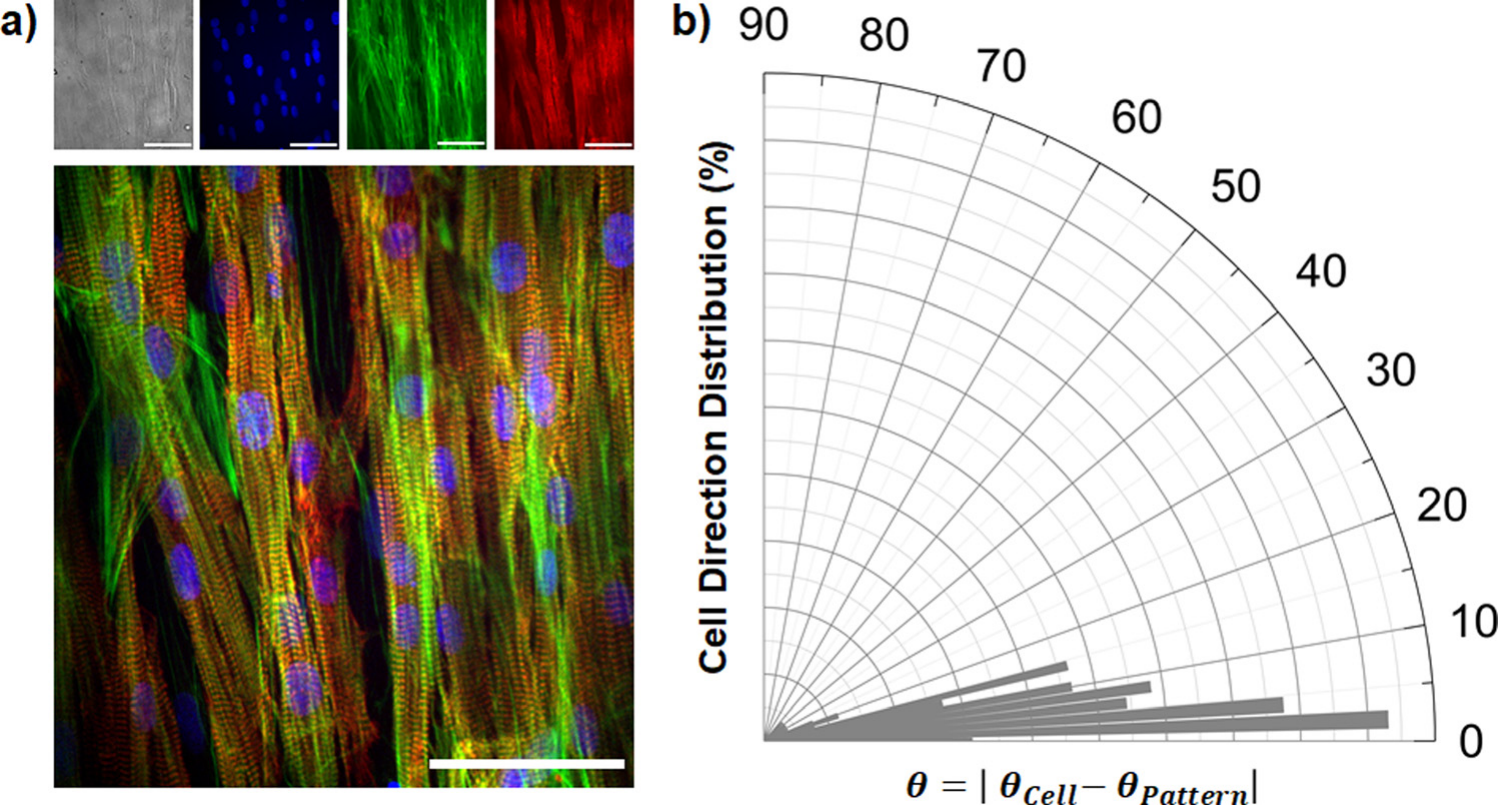
Engineering anisotropic cardiac tissue on micro-molded gelatin. (a) Fluorescence images of anisotropic NRVMs tissue (scale bar 20 *μ*m). The cells are stained with DAPI (blue), F-actin (green), and a-actinin (red). (b) Distribution of the cellular nuclear orientations on MTFs. The orientation distribution is normalized to the pattern direction (N = 130 nuclei from 3 MTFs) (average angle of the distribution, $\theta_{\mathrm{mean}}=8.89{}^{\circ}\pm 0.75{}^{\circ}$).

Initially, we assessed possible detrimental effect of the molecule on cell viability via 3-(4,5-dimethylthiazol-2-yl)-5-(3-carboxymethoxyphenyl)-2-(4-sulfophenyl)-2H-tetrazolium (MTS) assay. We treated neonatal rat ventricular myocytes (NRVMs) with Ziapin2 for 7 min followed by a Tyrode's solution washing step (complete internalization protocol is described in the supplementary material). We evaluated four conditions related to Ziapin2 internalization and light photostimulation (untreated cells in the dark, untreated cells exposed to light, Ziapin2-treated cells in the dark, and Ziapin2-treated cells exposed to light). In all the four conditions, there were no significant variations in the metabolic activity, confirming the negligible phototoxicity of the irradiation conditions as well as the low cytotoxic effects of Ziapin2 (Fig. 2). Once we excluded possible bio-compatibility issues, we focused on cardiac tissue seeded onto micro-molded gelatin MTFs. This cardiac chip is composed of a bottom adherent portion, which is attached to the underlying substrate and a series of partially detached cantilevers films capable of undergoing deflection upon cardiomyocyte contraction. MTFs were fabricated following previously reported protocols (Fig. S1).^72^ NRVMs were seeded on the MTFs, which promoted subsequent anisotropic tissue morphogenesis. Cell growth and anisotropy were then further evaluated by measuring the nuclei orientation relative to aligned groves of the underlying film (Fig. 3). This resulted in a clear anisotropic distribution, as evidenced by the small dispersion of the mean angle alignment, equal to $\theta_{\mathrm{mean}}=8.89{}^{\circ}\pm 0.75{}^{\circ}.$

**FIG. 4.**
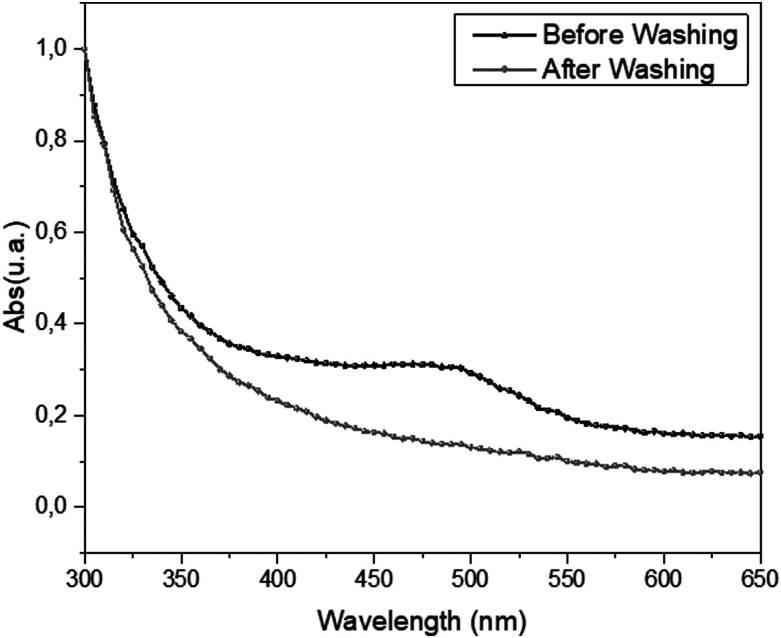
Assessment of the absence of adsorbed Ziapin2 onto gelatin film. Possible adsorption of Ziapin2 into the gelatin film was evaluated by comparing the film absorbance spectra upon exposure of gelatin to 5 *μ*M Ziapin2 for 7 min and after washing Ziapin2 with Tyrode's solution.

Finally, we made a last control more related to the photostimulation process. Due to the hydrogel-like nature of the gelatin substrate, we exclude possible adsorption of Ziapin2 into the substrate. We performed this test to also exclude possible unexpected storage of Ziapin2 into gelatin or thermal effect due to Ziapin2-druged substrate. This test was achieved by monitoring Ziapin2 optical absorption (Ziapin2 absorption band peaks at 470 nm) in an unpatterned molecule-treated gelatin film. The substrate was treated with Ziapin2 for 7 min following the internalization protocol described in the supplementary material. This protocol has been previously optimized for Ziapin2 cell membrane partitioning and, in this experiment, it has been conserved to mimic the molecules delivery into cell membrane. We observed the presence of Ziapin2 absorption peak during the 7-min treatment while no residual traces of the molecule were detected after the washing step (Fig. 4). This shows that that there is no significant Ziapin2 adsorption onto the gelatin films, avoiding unexpected effect during photostimulation process.

**FIG. 5.**
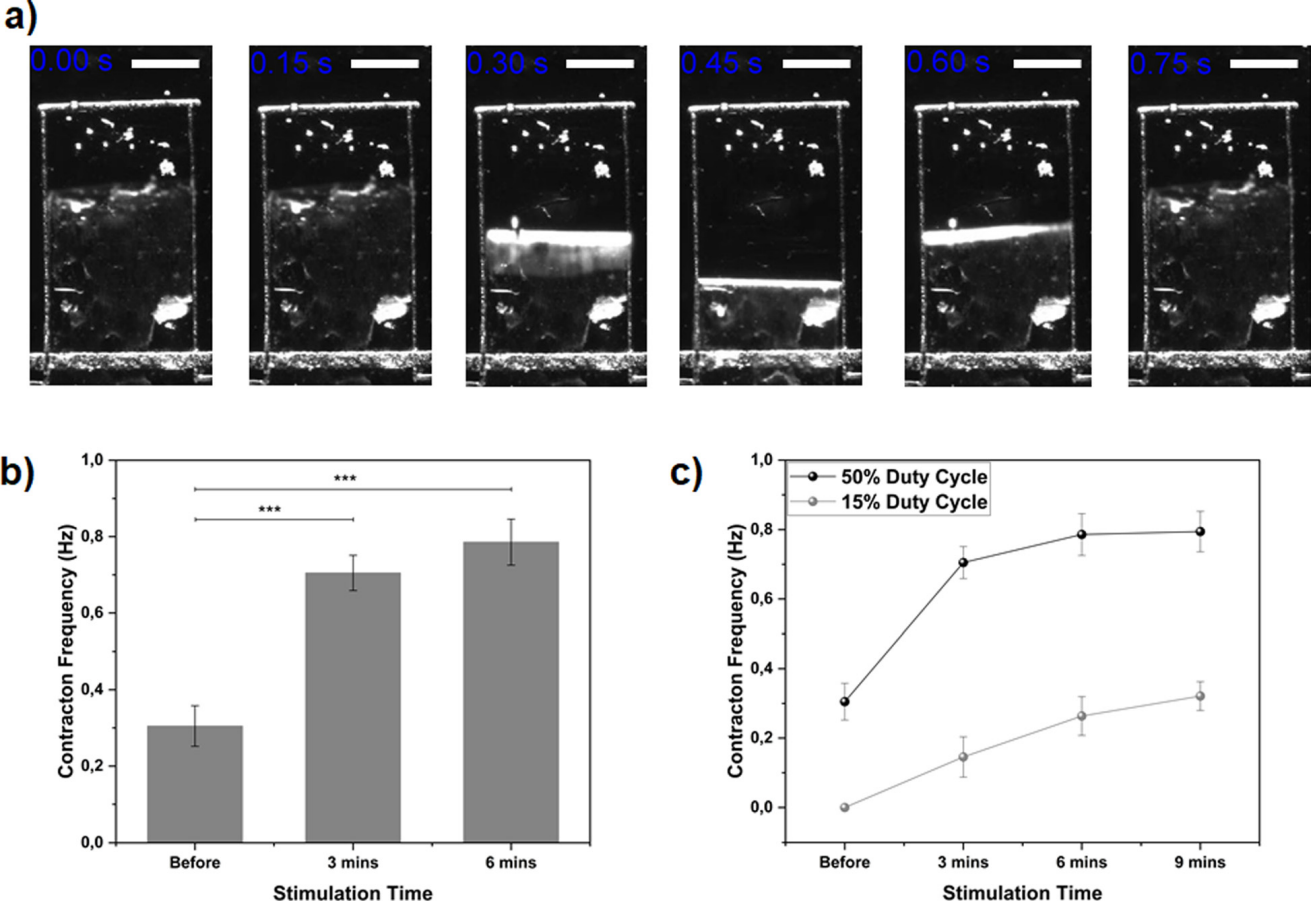
MTF photostimulation. (a) Time course representing the contractile activity of a photostimulated cantilever (scale bar 1 mm). (b) Histogram representing the contraction frequency for spontaneous activity (before, stimulation time = 0), and 1 Hz pulsed stimulation (3 and 6 min). Statistical significance in the figure is expressed as ^*^ for a p-value < 0.05, ^**^p-value < 0.01, ^***^p-value < 0.001. Data are represented as mean±standard error of the mean (s.e.m.). Ziapin2-treated MTFs are represented in light gray (N = 40 cantilevers from 8 MTFs) while untreated MTFs are reported in the dark gray (N = 11 cantilevers from 3 MTFs). (c) Effect of different light exposure patterns on the contraction frequency of the MTF. (Dark gray line duty cycle = 50% and light gray line duty cycle = 15%). Data are represented as mean±standard error of the mean (s.e.m.). N = 10 cantilevers from 2 MTFs.

**FIG. 6.**
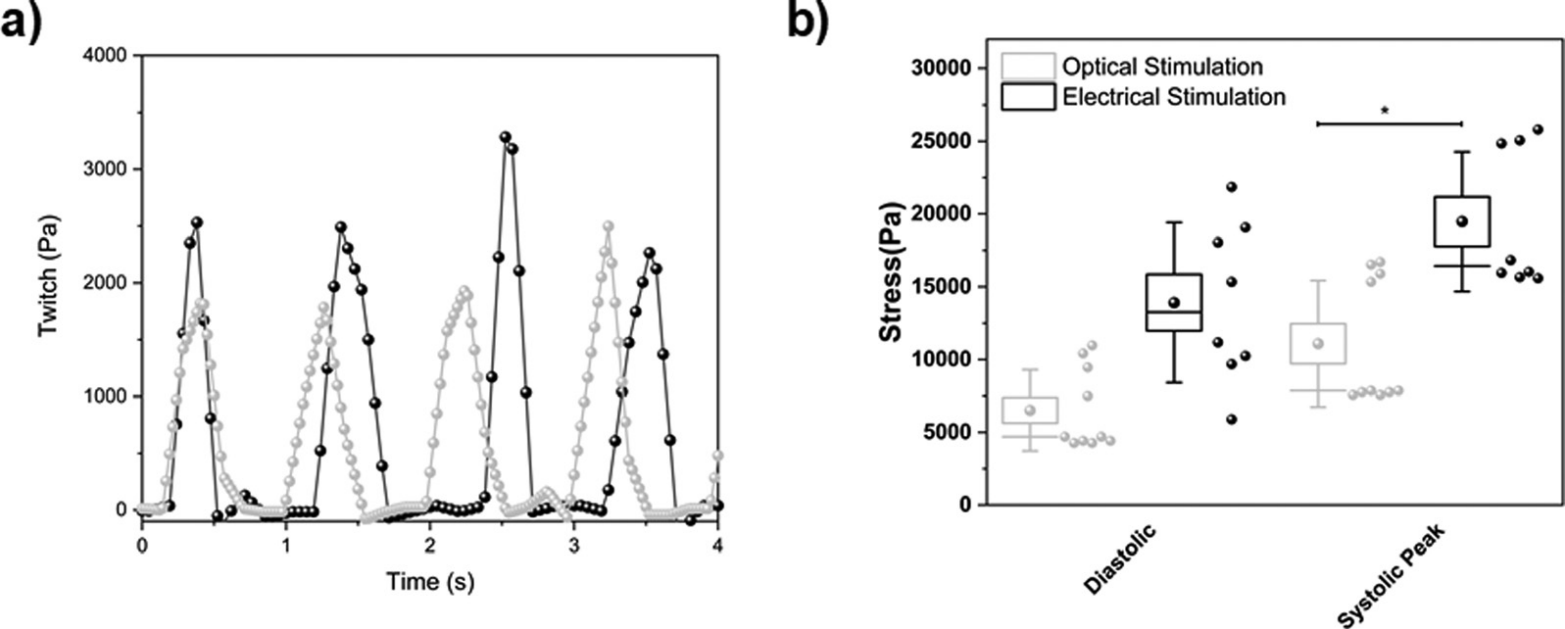
Extracted contractile MTF curves activity. Data extracted from the acquired video using a custom ImageJ script. (a) Representative stress traces for each stimulation methods. (b) Stress summary representing the diastolic and peak systolic stresses extracted for photostimulation (N = 10 cantilevers, 2 chips) and electrical stimulation (N = 8 cantilevers). Light gray color was used to indicate the photostimulated MTFs while dark gray was used for electrical-triggered MTFs.

Once we excluded possible unexpected interaction between materials and bio-compatibility issues, we evaluated the effects of Ziapin2 on cardiac tissue seeded onto micro-molded gelatin MTFs. Ziapin2's ability to pace cardiomyocytes was then tested by monitoring both the MTF's contraction and calcium wave generation. In each case, NRVMs were treated with Ziapin2 and placed in warmed Tyrode's solution. Contraction was quantified by recording the cantilever's motion (Figs. 5, S2, and S3) and using Stoney's equation to extract stress measurements. MTFs were stimulated by a protocol involving a 3-min pulse light excitation (at 1 Hz frequency, 60 mW/cm^2^) followed by a video recording without photostimulation (a graphical representation of the stimulation process is reported in Fig. S4). This stimulation protocol was developed to avoid optical interference resulting from the photopacing presence during video acquisition. Moreover, we set the duration of the acquired video to 30 s avoiding longer acquisition that could result in a decrease in the light-induced contraction activity. The results of the photostimulation (Fig. 5) show a clear increase in the MTFs contraction frequency. In the absence of optical stimulation, the mean spontaneous contraction frequency was 0.31±0.10 Hz. After two cycles of the described stimulation protocols (meaning 6 min of pulsed light stimulation), this value was increased up to 0.78±0.11 Hz [Fig. 3(b)] while no increase in contraction was detected in the absence of molecule [Fig. 3(b)]. We also evaluated the effect of this photopacing effect by reducing the stimulation duty cycle from 50% to 15%. The variation of duty cycle is directly connected to the effective light pulse length. In particular, a 50% duty cycle produces a 500 ms light pulse, while 15% cycle produces a 150 ms pulse without changing the stimulation frequency. We observed a lower pacing effect achieving a lower contraction frequency of the MTF cantilevers (final achieved contraction frequency is 0.8 Hz for 50% duty cycle and 0.3 Hz for 15%). Furthermore, we compared the effect of the electrical and optical stimulation in terms of MTF's stress traces (Fig. 6). We observed a lower value for both diastolic and systolic peak under optical stimulation while the average twitch stresses were approximately similar (4.8 kPa for electrical and 5.4 kPa for optical).

**FIG. 7.**
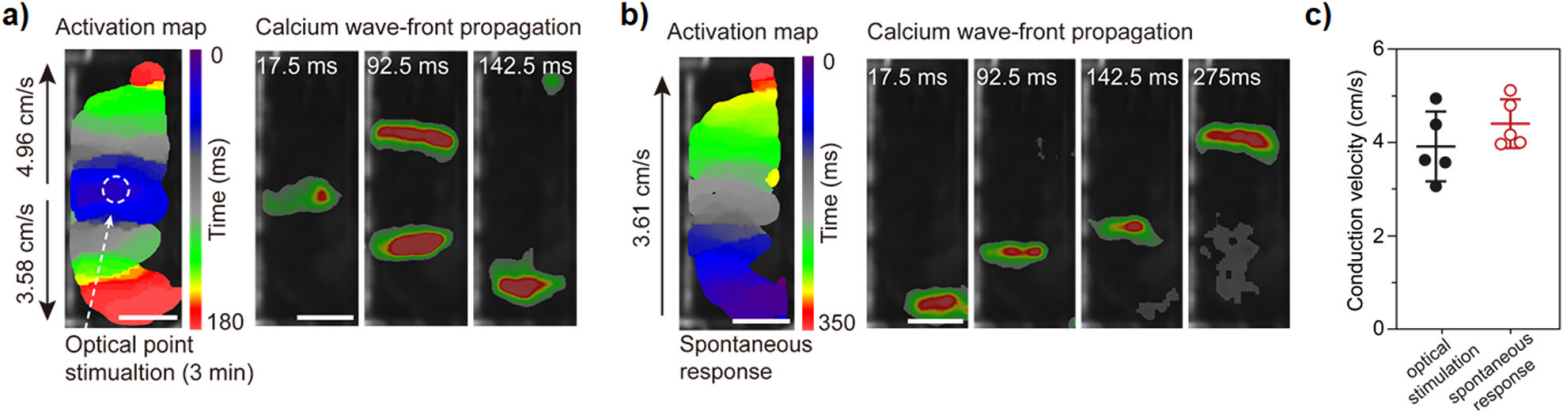
Optical mapping of light-sensitive azobenzene compound for activating cardiac tissue. Representative calcium wave triggered by Ziapin2-mediated photostimulation (a) and spontaneous activities of MTFs (b). In the photo-generated wave, the presence of a double wave front provides a clear indication of the Ziapin2-mediated photopacing. (c) Box plot of propagation velocity extracted by different cantilever (black dots refer to photogenerated calcium waves, while red dots represent spontaneous calcium waves; N = 3. 2 replicates per sample, scale bar = 2 mm).

Calcium wave generation and propagation was measured by optical mapping. This information was collected by stimulating the center of the Ziapin2-treated cantilever using an optical LED fiber and monitoring by using calcium indicators the calcium activity (Figs. 7, S5, and S6). These experiments show the generation of two propagating waves (originated by the light spot) for photostimulated cantilever while a single wave starting from the edge of the cantilever for the spontaneous wave. The spontaneous calcium wave average velocity is 4.2 cm/s along the cantilever.

Even though this velocity is slightly altered by the Ziapin2 presence, no significant variations were detected among spontaneous and light-activated waves (photo-generated waves average velocity is equal to 3.9 cm/s). In conclusion, we compared the level of reactive oxygen species (ROS) production due to the stimulation (both optical and electrical, 3 min stimulation at 1 Hz). This was measured in acute condition by using the CellROX assay (Figs. S7 and S8). We did not find any significant differences among the stimulation processes.

## III. DISCUSSION

In this paper, we demonstrate that tissue photopacing of a muscular film can be achieved optically by using Ziapin2 as a phototransducer. We show that Ziapin2 is neither toxic nor phototoxic, and it successfully partitions into the cardiomyocyte membrane and there it enables cell contraction once. Furthermore, we exclude any undesired effect coming from Ziapin2-gelatin interaction by demonstrating that there are no adsorbed molecules into a flat gelatin film at the end of a treatment process.

The MTFs response to photostimulation is clear, showing a significant enhancement of contraction activity under pulsed illumination. We also observe that the contraction frequency depends on the duty cycle, tightly linking the MTF contraction with the amount of delivered photons. The duty cycle dependence was unexpected, based on our experience with single HEK293 cells and neurons photostimulation where the pulse duration plays a secondary role on the membrane potential modulation. Differently, here shorter stimulation pulses (roughly the pulses length is reduced three times passing by 50% duty cycle to 15%) are less efficient, highlighting that there are complex phenomena, beyond single cell excitation, taking place during a tissue photostimulation. The complexity of the physiological process, which is indirect and involves a number of intermediate steps, could also explain why the photoinduced contraction rate stays lower than the stimulation rate. Another intriguing possibility arose from this duty cycle dependency. The duty cycle tuning (and relative reduced effect on the contraction frequency) offers an additional way to control the photostimulation efficacy. Indeed, these results suggest the ability to tune the achieved contraction frequency through the duty cycle, thus adding an innovative parameter as an alternative to light intensity and stimulation time. This can be open grounds for translational applications expanding the possible methods for achieving a specific final contraction frequency.

A further demonstration that Ziapin2 photoexcitation induces a physiological contraction of the MTFs comes from the calcium activity that is mechanistically associated with calcium waves within the tissue. This evidence corroborates the Ziapin2 photopacing effect showing that waves start at the stimulation point while spontaneous waves are generated at the edges of a cantilever where the source-sink is more favorable. This evidence confirms the control over the pacing of the cardiac tissue as well as the spatial selectivity of the proposed photostimulation method. Moreover, comparing the photoactivated waves with the spontaneous ones, we see that the propagation properties do not undergo significant variation in the presence of Ziapin2. We also attempted a comparison between optical and electrical stimulation analyzing the ROS production and measuring the cantilever stress traces. The CellROX assay does not show significant differences between the two stimulation methods highlighting a negligible cytotoxic effect during the photostimulation process. These are encouraging results considering both the absence of wiring and electrodes excluding possible hydrolysis effect. The absence of hydrolysis as well as other detrimental effects associated with electrode degradation promotes Ziapin2 as a valuable tool for a long-term stimulation of the microphysiological model. This condition could be exploited for drug testing, diseases development studies, and muscle aging process where the time evolution plays a crucial role. Moreover, this photostimulation approach does not require *in loco* device or electrode allowing the contemporary acquisition of microscopy images for tissue condition monitoring.

Furthermore, light allows the selective stimulation of a single cell allowing a specific targeting in a tissue. Regarding the extracted stress curves, we did not detect any significant changes in the diastolic value. In contrast, the systolic values were measured to be higher for electrical stimulation. These differences could be related to the amount of energy delivered by the two different techniques and by the different localization of the delivered stimulation. In particular, photostimulation works in a precise and localize point while the electrical one strongly depends from the geometry of the system as well as from the shape and dimension of the electrode. Anyway, considering the average value of the twitch, there are no significant differences between electrical and optical stimulation.

## IV. CONCLUSION

We successfully demonstrated the utility of a molecular phototransducer for pacing the contractions of an *in vitro* biohybrid actuator. The proposed photostimulation technique has a series of key enabling features such as being electrode-free, minimally invasive, genetic modification-free, and with high spatiotemporal selectivity. This first proof-of-concept opens up new possibilities for *in vitro* cardiac mechanistic studies as well as for soft robotics. Future developments will be focused on the realization of more complex bio-hybrid actuators that could perform light-driven tasks where the wiring absence advantage could be better exploited. Similarly, increasing the actuator complexity in synergy with the light space patterning could be exploited for realizing composite movements better mimicking physiological structure. Similarly, we will also evaluate possible *in vivo* application aiming at validating Ziapin2 as a valuable non-genetic tool. In this direction, we are also working for shifting the phototransducer absorbance toward less energetic radiation.

## V. MATERIALS AND METHODS

### A. NRVM isolation

The isolation of neonatal rat ventricular cardiomyocyte (NRVM) was performed based on previously published protocols (Park SJ, 2016; Feinberg A, 2007) and was approved by the Institutional Animal Care and Use Committee at Harvard University. Briefly, ventricles were isolated from two-day-old neonatal Sprague-Dawley rat pups (Charles River Laboratories, Wilmington, MA). The tissue was minced mechanically and rinsed in Hanks' balanced salt solution (HBSS; Thermo Fisher, Waltham, MA). This was followed by digestion in 1 mg/ml trypsin (MilliporeSigma, St. Louis, MO) in HBSS solution at 4 °C for 14 h, applying a gentle rocking motion to agitate digestion. The tissue was further homogenized using four digestion steps containing 1 mg/ml collagenase (MilliporeSigma, St. Louis, MO) in HBSS solution. Digestions were performed at 37 °C for 2 min each. After further gentle agitation by pipette, ventricular cardiomyocytes were collected from the solution by centrifugation and were filtered through a 40 *μ*m cell strainer to remove undissociated tissue. To remove excess fibroblast and endothelial cells, a prelating step was used. Pre-plating consisted of a 2 h and 15 min culture in a T175 flask, where fibroblasts preferentially adhered to the substrate. Next, cardiomyocytes were resuspended in culture media and re M199 media (Life Technologies, Carlsbad, CA) supplemented with 10% (v/v) heat-inactivated fetal bovine serum (Life Technologies, Carlsbad, CA), 10 mM HEPES (Life Technologies, Carlsbad, CA), 1% (v/v) MEM non-essential amino acids (Life Technologies, Carlsbad, CA), 3.5 g/l glucose (MilliporeSigma, St. Louis, MO), 2 mM L-glutamine (Life Technologies, Carlsbad, CA), 1.5 *μ*M/l vitamin B12, and 50 U/ml penicillin (Life Technologies, Carlsbad, CA). Cardiomyocytes were counted, adjusted to seeding density and ready to be seeded. After 24 h incubation, the substrate was washed with PBS to remove non-adherent cells and provided with fresh 10% FBS media. After another 24 h, we switched the media to M199 media supplemented as above but with 2% FBS to minimize growth of fibroblasts. Subsequently, the media was replaced every 48 h until use, typically within 3 to 5 days, but no more than 6 days post seeding.

### B. Muscular thin film (MTF) fabrication

MTFs were fabricated by micro-molding gelatin onto glass substrates, which were then seeded with neonatal rat ventricular myocytes (NRVMs). Fibronectin coatings were used to promote cell attachment, forming confluent anistropic tissue monolayers. This architecture provides support to the MTF and allows for the detection of photoinduced macroscopic motion. A 1 × 1 in.^2^ acrylic substrate was first covered with a protective film tape (Patco 5560 Removable Protective Film Tape). We used a laser engraver (30 W, 24 × 12; Epilog Laser Mini, CO, USA) to cut the tape for defining low adhesion and high adhesion regions for the gelatin layer as shown in Fig. S2(a). The tape was first peeled from the high adhesion region (i.e., bottom part of the acrylic substrate) to expose this surface for plasma oxygen treatment for 2 min. This treatment allows the bottom portion of the gelatin to adhere more onto the substrate than the top part that is dedicated for cantilevers. The top part of the tape was removed prior to gelatin deposition. The gelatin (10% w/v) and microbial transglutaminase (4% w/v pre-warmed at 37 °C) solutions were mixed at 60 °C and then were immediately dropcasted onto the pretreated substrate described above. A Polydimethylsiloxane (PDMS) stamp templated with the desired line pattern was then used to press the gelatin mixture while curing at room temperature. After curing, the PDMS stamp was carefully lifted from the gelatin surface and the laser engraver was used to cut 3 × 4 mm^2^ rectangular cantilevers (on the low adhesion region).

### C. Ziapin2 Administration

Ziapin2 was synthesized from the commercial Disperse Orange 3, according to a previously published procedure. It was administered to NRVMs following a previously reported protocol for photoinducer uptake, where cells were observed to display energy independent membrane integration and uptake. Here, cells were exposed to 5 *μ*M Ziapin2 for 7 min. This was consistent with previous reports, where it has been shown through time-lapse confocal microscopy that the signal of Ziapin2 increased in fluorescence intensity for up to 7 min, with longer exposure time minimally impacting further internalization. Subsequent to exposure, a washing step was performed to remove the excess of reagents remaining in the delivering medium. All procedures for Ziapin2 exposure were performed using Tyrode's solution (1.8 mM CaCl_2_, 5 mM glucose, 5 mM HEPES, 1 mM MgCl, 5.4 mM KCl, 135 mM NaCl, 0.33 mM of NaH_2_PO_4_, pH 7.4), including both delivery and washing buffers.

### D. MTS assay

The metabolic activity of NRVMs exposed to Ziapin2 was evaluated using an MTS assay (abcam ab197010). This test is based on the reduction of the MTS tetrazolium compound, approximating total cell viable. This reduced form of the formazan dye is characterized by an absorbance at 500 nm. The reagent (10% v/v in culture medium) was added to NRVM plated on a gelatin flat film placed in a 24 multi-well plate and incubated for 30 min. The plates were then measured using a plate reader.

### E. MTF contraction analysis

Gelatin MTF substrates with engineered cardiac tissues were transferred to a 35 mm Petri dish containing approximately 4 ml of Tyrode's solution (1.8 mM CaCl_2_, 5 mM glucose, 5 mM HEPES, 1 mM MgCl, 5.4 mM KCl, 135 mM NaCl, 0.33 mM of NaH_2_PO_4_, pH 7.4). The dish was placed on the stage of a Leica MZ9.5 stereomicroscope (Wetzlar, Germany). Throughout the duration of the experiments, a thermostat was used to maintain physiological temperatures. Optical stimulation (1 Hz frequency and 60 mW/cm^2^) was performed using a Spectra X light Engine (Lumencor). The light pattern was applied for 3 min before the acquisition of video. Electric stimulation was performed using platinum electrodes inserted into the dish and applying a signal with 1 Hz frequency and 5–7 amplitude using a MyoPacer Cell Stimulator (IonOptix, Milton, MA). MTF's cantilevers were recorded at 100 frames per second using a Basler A601f-2 camera (Exton, PA) right after the photostimulation or during electrical stimulation. To convert movies to stress measurements, movies were thresholded, and the radius of curvature for each MTF was calculated using the *x*-projection and original length. The radius of curvature, thickness, and elastic modulus of each MTF was used to calculate stress using modified Stoney's equation. For each MTF, the average diastolic and systolic stresses were calculated, averaged, and compared using Student's t-test.^66,75^

### F. Optical mapping

After 4 days in culture, NRVMs seeded on gelatin-based tissue chips were incubated with 2 *μ*M X-Rhod 1 for 30 min at 37 °C and then incubated in dye-free media for 5 min for washing. After the staining and washing, chips were kept in media for 10 min prior to the Optical Mapping System (OMS) run and then placed in Tyrode buffer (1.8 mM CaCl_2_, 5 mM glucose, 5 mM Hepes, 1 mM MgCl_2_, 5.4 mM KCl, 135 mM NaCl, and 0.33 mM NaH_2_PO_4_ in de-ionized water, pH 7.4, at 37 °C; Sigma). Calcium propagation was monitored using a modified tandem-lens macroscope (MiCAM Ultima, Scimedia) equipped with a high-speed camera (MiCAM Ultima, Scimedia), a plan APO 0.63× objective, a collimator (Lumencor), and a 200 mW Mercury lamp (X-Cite exacte, Lumen Dynamics).^17^ Recordings were acquired at a frame rate of 400 frames per second, and tissue chips were placed in a temperature-controlled Petri dish and imaged from above. The optical point stimulation was applied at one end of the tissue using an LED light source (465/25 nm, Doric Lenses). Pacing frequency was 1 Hz for 3 min (with a custom LabVIEW program; National Instruments) and was generated using optical fibers at a distance 1 mm from the tissue. Post-processing of data was conducted with custom software written in MATLAB (MathWorks). A spatial filter with 3 × 3 pixels was applied to improve the signal/noise ratio. Then, the conduction velocity of each pixel and each pulse was determined by calculating the x- and y-directional change rate.

### G. Immunofluorescence imaging

MTFs were fixed with 4% paraformaldehyde and 0.25% Triton X-100. NRVM's tissues were stained with monoclonal mouse anti-(sarcomeric α-actinin) primary antibody (Sigma), DAPI (Sigma), and phalloidin conjugated to Alexa-Fluor 488 (Invitrogen). Samples were then imaged using Olympus IX-83 spinning disk confocal microscope (Olympus) and recorded on an Orca Flash 4.0 C11440 (Hamamatsu) camera at 16-bit depth, with a 0.16 to 0.33 *μ*m pixel resolution.

### H. CellROX assay

The oxidative stress generated by optical and electrical stimulation was measured by a fluorogenic CellROX^®^ green probe (Invitrogen; Carlsbad, CA). Four different groups of NRVMs were seeded on 24-well plates. One group was photostimulated for 3 min using 1 Hz light pulse. The second group was electrically stimulated using the same frequency. Finally, a negative (untreated cells) and positive [treated with 200 *μ*M menadione for 1 h (Sigma-Aldrich, St Louis, MO, USA] control were performed with the third and fourth group. Cells, after stimulation and menadione treatment, were incubated in 5 *μ*M CellROX^®^ green and 20 ng ml^−1^ Hoechst stain (ThermoFisher Scientific, Waltham, MA, USA) for 30 min. Samples were rinsed with PBS, fixed in 4% PFA, and imaged immediately using an EVOS M7000 Imaging System (ThermoFisher Scientific, Waltham, MA, USA). CellROX^®^ intensity values were normalized to the number of Hoechst-positive cells and corrected for background fluorescent intensity of cells and NRVMs without CellROX^®^.

### I. Statistical analysis

Data are represented as mean±standard error of the mean (s.e.m.). Statistical significance between two conditions was evaluated using Student's t-test. In all the reported data, ^*^P < 0.05, ^**^P < 0.01, ^***^P < 0.001.

## SUPPLEMENTARY MATERIAL

See the supplementary material for a graphical representation of the MTFs' fabrication steps (Fig. S1). A representative video of the spontaneous (Fig. S2) and the photoinduced (Fig. S3) MTF contraction activity. A sketch of the photostimulation protocol (Fig. S4). A video of photoactivated and spontaneous (Figs. S5 and S6) calcium wave on MTF. CellROX control experiment (Fig. S7) and comparison between optical and electrical stimulation (Fig. S8).

## Data Availability

The data that support the findings of this study are available within the article and its supplementary material.
